# A Creative Communication Partnership to Promote Curricula Dissemination on Social Media

**Published:** 2024-02-21

**Authors:** Atom J. Lesiak, Natasha Malik, Joan C. Griswold

**Affiliations:** 1 Genome Sciences Education Outreach, Department of Genome Sciences, University of Washington, Seattle, WA; 2 CommLead, Department of Communications, University of Washington, Seattle, WA

**Keywords:** Teacher PD, Communication, Social Media, Campaign, Teacher Networks, Curriculum Dissemination

## Abstract

Genome Sciences Education Outreach (GSEO) has developed innovative programs that bring leading-edge science to teachers and students in K-12 schools. Disseminating educational materials equitably and accessibly to teacher stakeholders to maximize reach and impact is challenging for many programs. Traditionally, programs connect materials with teachers through local networks, in-person professional development sessions, and at regional and national conference presentations. The need for curricular changes in 2020 spurred the proliferation of online and digital educational materials and professional development opportunities. These digital materials—now available to a worldwide audience—require a shift in dissemination strategy to enhance the potential reach of these materials both locally and nationally. This manuscript reports a case study of a dissemination approach, to create a collaboration between GSEO and CommLead (the communications master’s program at the University of Washington) to promote education materials developed by the publicly-funded Genes, Environment and Me Network (GEMNet) program. This manuscript describes the development and the ad hoc implementation and evaluation of a social media campaign to expand the reach of the GEMNet curricula. With a targeted social media campaign on Facebook, GSEO was able to dramatically and affordably increase the reach of the GEMNet curricula and expand the potential impact and utilization of educational materials to a nationwide teacher audience, highlighting the potential for other similar collaborations to efficiently enhance the dissemination strategy of other education outreach programs.

## INTRODUCTION

### Curricula Dissemination.

Education outreach programs that create pedagogically sound curricula and educational materials for teachers have a long history of improving education outcomes for students. (Morgan, 1977; Paul Westmeyer, Earl Brakken, 1967). The primary method for programs to disseminate educational materials to teachers has focused primarily on in-person teacher workshops and professional development trainings on-site or at teacher conferences. Access to professional development opportunities are limited by teacher capacity (time, energy, money, family responsibilities) and restrictions regarding state and district-mandated standards (Craig, 2006). Notably, rates of teacher implementation of curricula after short awareness workshops where lessons are presented briefly to teachers rather than covered in detail have been identified as being just as effective as longer, in-depth, implementation workshops (Mayer and Fortner, 1987). Thus, other less intensive strategies for dissemination that simply raise teacher awareness of materials, such as mailed newsletters, magazines, websites, and e-newsletters, have an appreciable potential to provide accessible materials to teachers more equitably and efficiently than in-person workshops (McBeath, 1997; Stewart, Martyn and Thompson, 2005).

### Digital Dissemination on Social Media.

Social media platforms, like Twitter, Facebook, and Instagram, have emerged as tools for teachers and education outreach programs to build community, network, and share information (Al-Qaysi et al., 2023; Carpenter et al., 2020, 2023). Although some programs have been successful in using social media for connecting with students in rural areas, there remain important challenges related to teacher-student engagement on social media platforms (Carpenter et al., 2019; Carpenter and Harvey, 2019; Chester et al., 2018). Social media is perhaps better suited for connecting adult professionals with resources, as teacher networks on social media platforms have emerged as a hub for teachers and education outreach programs to share professional materials and training opportunities with one another (Carpenter et al., 2023; Davis and Yi, 2022; Torphy et al., 2020). Advances in distance learning, online face-to-face conferencing, and the ability to develop online training modules, now offer an opportunity for education outreach programs to enhance their dissemination by engaging teachers online. Dissemination is no longer constrained by geographical limitations and can be enhanced by practices that utilize modern tools to connect teachers with training opportunities and resources.

### Marketing and Communications for Education Outreach.

For many curricula producing education outreach programs, finding effective and efficient ways to expand the reach of educational materials to a broader teacher population can be a challenge. The most reliable ways to connect with teachers is generally through presenting at teacher conferences or promoting Professional Development (PD) opportunities in regional schools; however, conference travel is often expensive with no attendance guarantees. Therefore, programs looking to promote their materials could benefit from utilizing marketing and communications strategies geared towards teachers. Promotion of materials to the target audience can utilize a lot of the strategies related to the field of science communication. Effective science communication involves an interdisciplinary skill set, requiring the ability to communicate a clear message and provide relevant information to stakeholders in an accessible and inclusive way (Borowiec, 2023; Collins et al., 2016; Haywood and Besley, 2014). Social media greatly expands the reach of communication beyond in-person venues, and opens up affordable marketing tools for education outreach programs. Integration of science communication strategies with marketing research trends for teachers could prove useful for education outreach programs looking for efficient and effective ways to disseminate their materials.

### Background.

Genome Sciences Education Outreach (GSEO) has developed innovative programs that bring leading-edge science to teachers and students in K-12 schools. Historically, GSEO has been successful disseminating lessons and curriculum materials at teacher conferences and local teacher PD workshops. Paying for teacher travel for on-site workshops and for GSEO staff to travel to teacher conferences was supported by funding from the NIH Science Education Partner Award (SEPA). With SEPA funding, GSEO created the Genes, the Environment, and Me Network (GEMNet) curricula for high school science, health, and family and consumer science (FCS), and disseminated it to teachers nationwide (Griswold and Lesiak, 2020). Program staff quickly identified that not all teachers had equal access to conference attendance and travel to Seattle for PD trainings. Even before the switch to online learning during the COVID-19 pandemic in 2020, GSEO began providing online PD in hopes of reaching more teachers, particularly those with limited capacity to travel on-site (Lesiak et al., 2021). GSEO has a long history of re-engaging teachers via their mailing list following engagement at conferences or on-site PD sessions, but lacked a strategy to effectively grow the reach of the GEMNet curriculum outside of those in-person networking spaces. As curricular needs changed during the COVID-19 pandemic, GSEO transitioned the curricula to a more accessible digital format to support online learning for both students and teachers, creating a product that could be widely disseminated nationwide.

During a programmatic self-assessment, GSEO identified that their ability to expand their teacher network was inhibited by their limited online presence and networks outside Washington state. Although considerable resources were dedicated to in-person dissemination workshops and curricula assessment, there was no specific funding to support program dissemination in digital spaces. Funding only provided financial support for GSEO’s two part-time team members to create and disseminate curricula, and execute a formal program evaluation. Neither team member had experience launching a targeted digital dissemination campaign online or on social media, and federal grant or departmental funds were not dedicated towards this effort. Effective program dissemination in digital spaces was limited by the lack of both the resources dedicated to digital marketing and a communications strategy to reach the target audience.

One of the limited benefits of travel restrictions and online conference attendance in 2020–21 was that GSEO was able to re-allocate budget surplus towards digital dissemination and communication strategies. These extra funds, around 6 thousand dollars, were insufficient to hire full-time staff to develop a communications plan. Prices for external contractors varied greatly based on experience level (from $20-$350 per hour), as did the number of hours required to complete the scope of work requested. Therefore, it was determined that hiring an external social media manager was not cost effective for GSEO’s current budget situation. Instead, alternative methodologies were employed to develop an interdisciplinary partnership with the University of Washington’s communications graduate program to hire a communications graduate student to promote GSEO’s educational materials.

### Case Study Framework.

This case study describes a curriculum dissemination approach via the creation of an on-campus collaboration to enhance GSEO’s communication strategy. At each phase of development and execution, an ad hoc implementation and evaluation of digital communication and social media strategies was utilized. Importantly, the description and analysis of this case study is intended to provide recommendations for other education outreach programs, and was not intended to be a formal, well-controlled, study of communication and dissemination strategies.

## METHODS

### Establishing a Novel Communications Collaboration On-Campus for Education Outreach.

With an external contractor beyond the scope of their budget, GSEO sought creative solutions to remedy their communication deficits by employing an inter-departmental cross-training partnership in communication and education outreach. Previous efforts to establish relationships with the University of Washington (UW) Communications Department, to establish an interdisciplinary partnership for undergraduate students to get science communication training, was unproductive. Eventually, GSEO reached out to CommLead, the communications master’s program at UW, and applied to be one of their community partners for the Fall 2021- Spring 2022 school year. In the partnership, GSEO would serve as a client by employing a communications masters student for ~$2000 per quarter or $15/hour. The masters student paired with GSEO could get project support from faculty in the communications department while gaining experience and training in science education by working for GSEO. There was no direct interaction between GSEO and faculty in the communications department, outside of quarterly check-in meetings with the CommLead program coordinator. In Fall 2021, co-author and graduate student Natasha Malik became the Communications Coordinator (CC) for GSEO based on a mutual interest in diabetes education.

### Development of the Communications Strategy.

Finding effective digital marketing strategies to reach teachers was determined to be the most effective way to support broader dissemination of the GEMNET curricula. In the literature, GSEO identified recurring strategies for effective marketing to teacher populations (Centers for Disease Control and Prevention, 2017; Chaffey and Bosomworth, 2013; MDR, 2023; Royle and Laing, 2014; Sheer ID, n.d.; The Influence Crowd, 2021; Tobey and Manore, 2014).

- **Understand your audience’s needs and desires.** It was identified that teachers may be interested in the ready-to-use Next-Gen Science Standard and Health standard-aligned curricula developed by the GEMNet program. Additionally, the detailed teacher instructions, both in video and written formats, can help teachers quickly implement the lessons in their classroom. Effective marketing strategies for teachers often include paid professional development and STEM clock hours, which were both previously supported by the SEPA grant funding for GEMNet, but not included as an element of the final communications campaign.
- **Identify where to reach your audience.** Teachers have an increased online presence relative to the general population, especially on social media platforms. The pros and cons of each social media platform were discussed before choosing a platform on which to focus the social media campaign (Barnhart, 2023; Bottles and Sherlock, 2011; Zhou, 2023).
- **Facebook (FB)** – 2.96 billion users, largest age bracket 25–34 and demographics skew older. Provides structure for community engagement with Groups feature, and there are many active national, state, and local teacher Group Pages. FB has an integrated Ad Center for targeted program promotion, and ability to “Boost” posts for a marginal fee.
- **Instagram** – Over 1 billion users, average age 25–34 and demographics skew younger. Utilizes hashtags # for targeted posts, integrated with Facebook, but no Group Page features.
- **Twitter** – 0.23 billion users, largest age group 25–34 with demographics skewing older and more educated. Uses hashtag (#) for targeting, and advertising options for tweet promotion.
- **TikTok** – 1 billion users, largest age group 18–24 skewing younger. Allows video posts only and limited external linking to websites.
- **LinkedIn** – 0.8 billion users, largest age group 25–34. Professional networking platform with integrated advertising features, but without groups feature.
- **Make a plan.** Language, messaging, timing, and eye-catching posts are critical. A plan to create posts highlighting key features of the free-to-use and standards-aligned GEMNet lessons was formulated. Facebook was chosen at the primary social media platform because of the integrated ad center, group page feature, largest user base, and average teacher age aligning with the most common age range of Facebook users (Statistics, 2012). The plan was to broaden the GSEO teacher network through email communication and combine this effort with joining other professional teacher group pages on Facebook, concluding with a social media campaign launched in the Spring.
- **Invest in Social Media.** Effective engagement on social media involves regular posting, and follow-up with audiences. Engagement includes responding to comments, sharing additional resources, and more. Paid advertisements can target specific demographics and groups. Paying to “boost” posts can expand the reach of posts, by sharing it with the “friends” of your followers. Investing in social media not only requires time to curate, engage, and post, but to expand your network. It also involves budgeting for paid promotions. During the described campaign, GSEO did not pay to “boost” posts or post advertisements, but have since paid to boost posts in 2023 to expand the reach of promotions for online PD.
- **Act and engage with the audience**. The Communications Coordinator followed-up with commenters, responded to additional requests, and regularly made posts during the campaign.
- **Measure, monitor, and evaluate**. The primary measure of a successful campaign was to measure traffic to the GSEO curricula website hosting the GEMNet curricula (Griswold and Lesiak, 2020). Google Analytics was utilized to track site visits before, during, and after the campaign. Additionally, the Facebook ad center provided information regarding reach of a post (how many people saw it on their page) as well as well as engagement (likes, comments, and shares).

Each quarter GSEO decided to focus on a different element of the communications objectives.

- Quarter 1 (Sept.-Dec. 2021): Conduct research on teacher associations in different states nationwide.
  - Contact teacher associations to determine posting in teacher organization websites and newsletters.
  - Connect on social media with teacher organizations.
- Quarter 2 (Jan.-March 2022): Develop advertisements for the GEMNet curriculum, and place in locations teachers will see them, to build off of Quarter 1.
- Quarter 3 (April-June 2022): Launch major social media campaign for all materials developed.

## RESULTS

### Communication Collaboration Outcomes.

#### Quarter 1 (Sept.-Dec. 2021).

During the first quarter of the collaboration, the CC found contact information and reached out to 75 different science teacher and health/FCS teacher associations nationwide via email to open communications about posting ads for the GEMNet curriculum on their webpage, publications, or newsletters. There was a focus on state chapters of teacher organizations because advertising with national teacher organizations like NSTA, NABT, and SHAPE America were cost prohibitive, with both digital and paper ads costing $1000 or more per post. In total, 41 regional science chapters of NSTA or NABT and 34 Health/FCS state chapters were contacted. The ask was that organizations share the GEMNet curriculum with teachers, either through newsletters or posts to web and social media pages. Email responses from state chapters of teacher organizations was minimal, with responses from four of the science teacher organizations contacted. Posts were made to the GEMNet curriculum resources in the newsletters of all the organizations that responded, and two offered to host a small posting/advertisement on their website free of charge. Unfortunately, the outcome of these posts was difficult to measure since there was no confirmation of posting.

More success was found in using Facebook to connect with local and state teacher organizations. Through the GSEO Facebook account, GSEO was able to join the group pages of nine of the 75 teacher organizations reached out to through email messages. Many of the state chapters did not have active Facebook groups; however, GSEO eventually posted on 8 science teacher organizations and one Health/FCS group. Each of the teacher organization group moderators encouraged posts advertising the GEMNet curricular resources on all the group pages joined. Page moderators generally review posts before they appear on the teacher site group pages. GSEO has respectfully limited posts on group pages to once per week (typically less than once per month) and refrained from re-posting the same materials more than once. Other social media platforms were considered but it was determined that teacher group pages, Facebook ad center, and the Facebook platform interface would be the most compatible with the campaign goals.

#### Quarter 2 (Jan.-March 2022).

**Figure 1.**
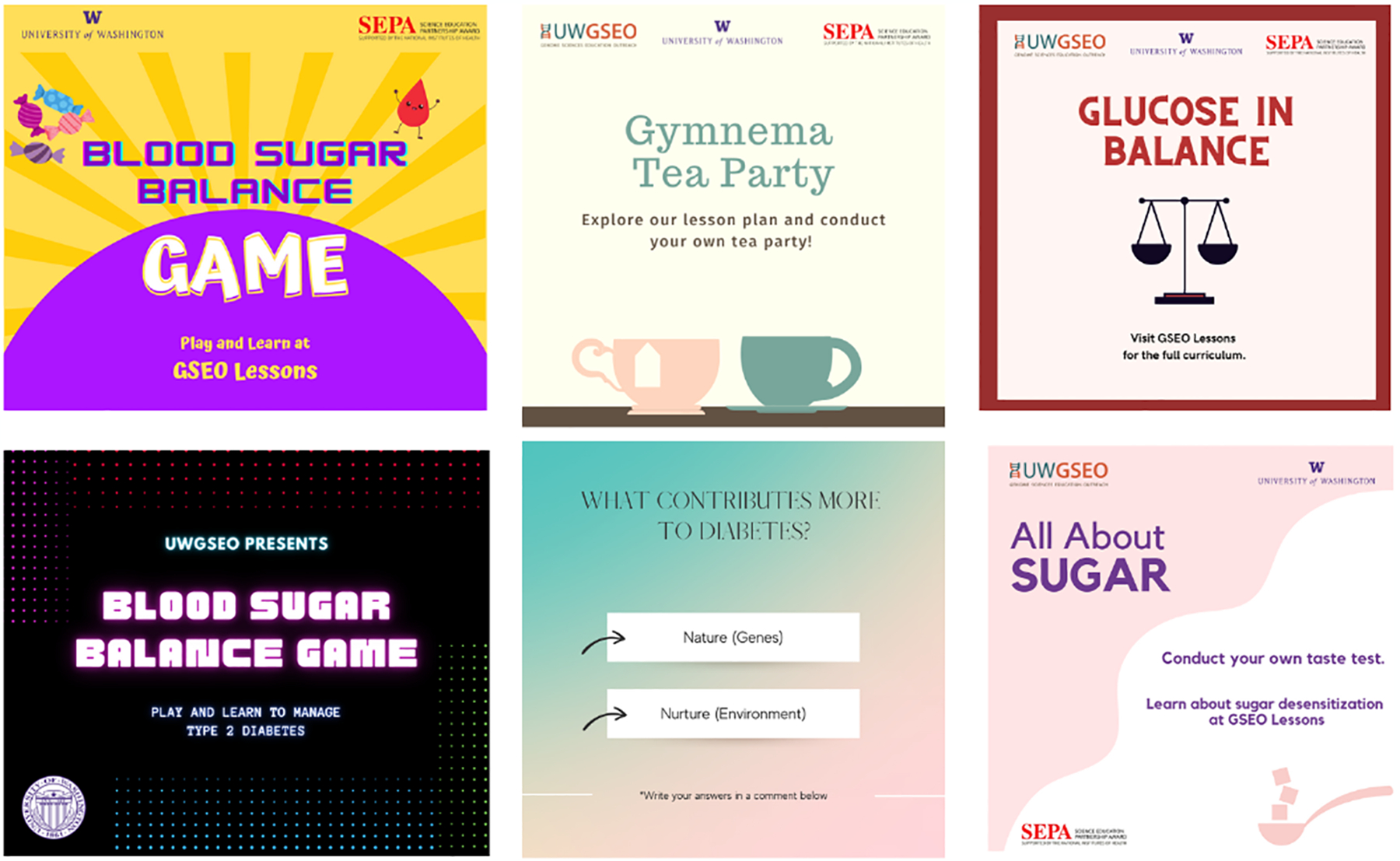
Example social media posts to promote lesson resources.

The GSEO CC utilized her communications and design skills to develop an array of advertisements for the GEMNet curriculum resources (Figure 1). Each ad utilized a specific color scheme and theming related to the content. Ads directed teachers to the primary curriculum hosting website on Google Sites (Griswold and Lesiak, 2020). This site was initially developed to support the online version of the curriculum and lessons, and served to support the online PD training sessions as well as presentations at national conferences. Teacher recruiting for online PD primarily utilized the GSEO listserv, developed over the years of outreach by the program (617 total subscribers). Promotion of presentations at national conferences was usually limited to a social media post made or listserv email sent in the weeks before the conference.

**Figure 2.**
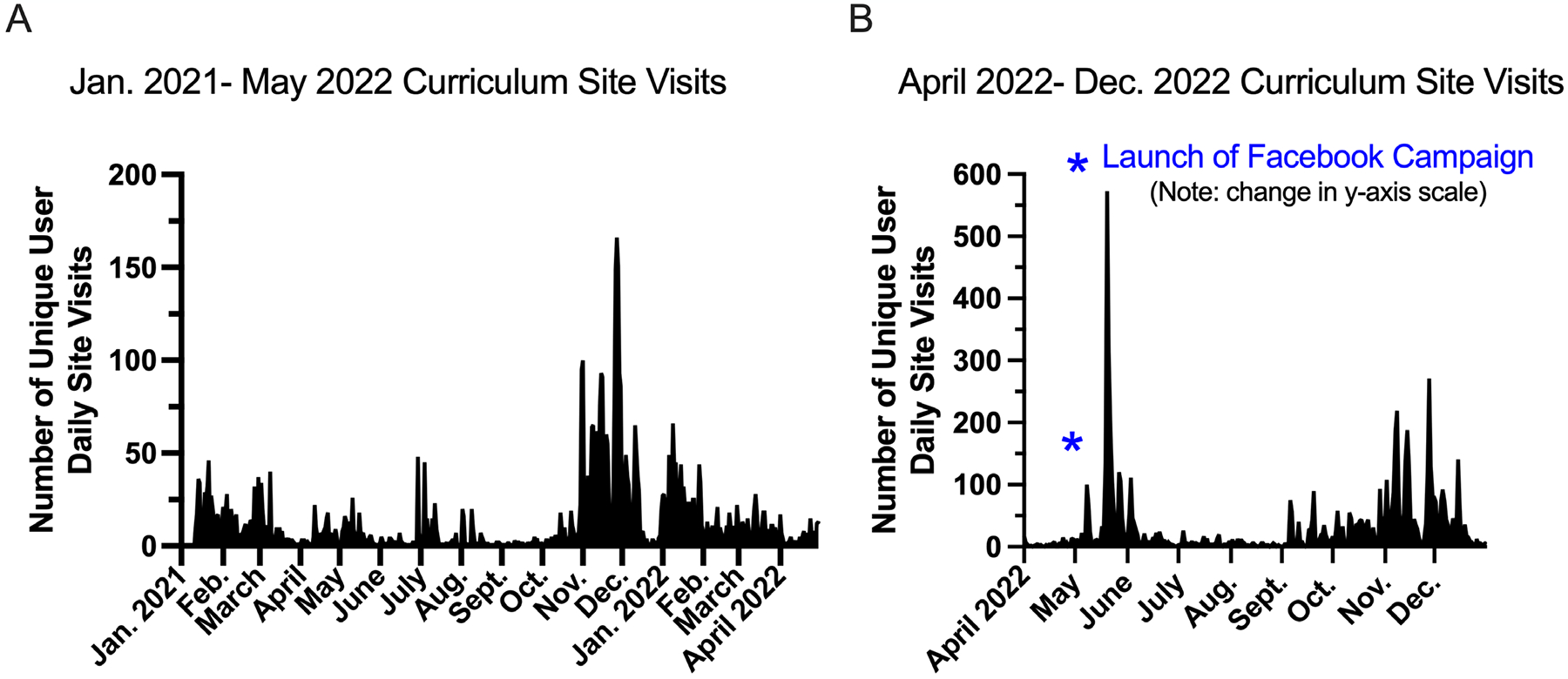
A) Unique user curriculum site visits from January 2021 to May 2022. B) Unique user curriculum site visits from April 2022 to Dec. 2022. *Date of Facebook Campaign launch.

Using Google Analytics, GSEO was able to measure site traffic to the GEMNet curriculum website since its creation in early 2020. During online PD and after teacher conference presentations there are regular upticks in site visits and engagement (Figure 2A). Therefore, GSEO was poised to compare curriculum website visitations during traditional activities to curriculum website visitations during the social media campaign with CommLead (campaign launched May 2022, Figure 2B). Metrics and analysis of data from this campaign was not designed to be quantitative research. Implementation and evaluations occurred extemporaneously during the study period. Data is provided to inform future program activities and communication strategies and not intended to provide quantitative, well-controlled, evidence regarding social media and communications strategies.

#### Quarter 3 (April-June 2022).

During the last quarter of the collaboration, the CC launched the social media campaign by posting ads for each of the GEMNet lessons on the GSEO Facebook page, as well as to a variety of teacher organization pages on FB (Figure 1). Before the campaign, bursts of 20–150 daily site visits were seen from Jan. 2021 through April 2022, with regular site visitations during the school year and periodic decreases during the summer and early fall (Figure 2A). Upon launching the FB campaign in May 2022, there was a dramatic increase in daily site visits, with daily site visits jumping to more than 500 visits per day. The biggest increase in site traffic followed posts about the diabetes game Blood Sugar Balance in late May 2022 (Figure 2B). Site visits dipped again at the end of the campaign in June and throughout the Summer; however, site visit numbers in Sept-Nov. 2022 were higher than that of the previous year.

**Figure 3.**
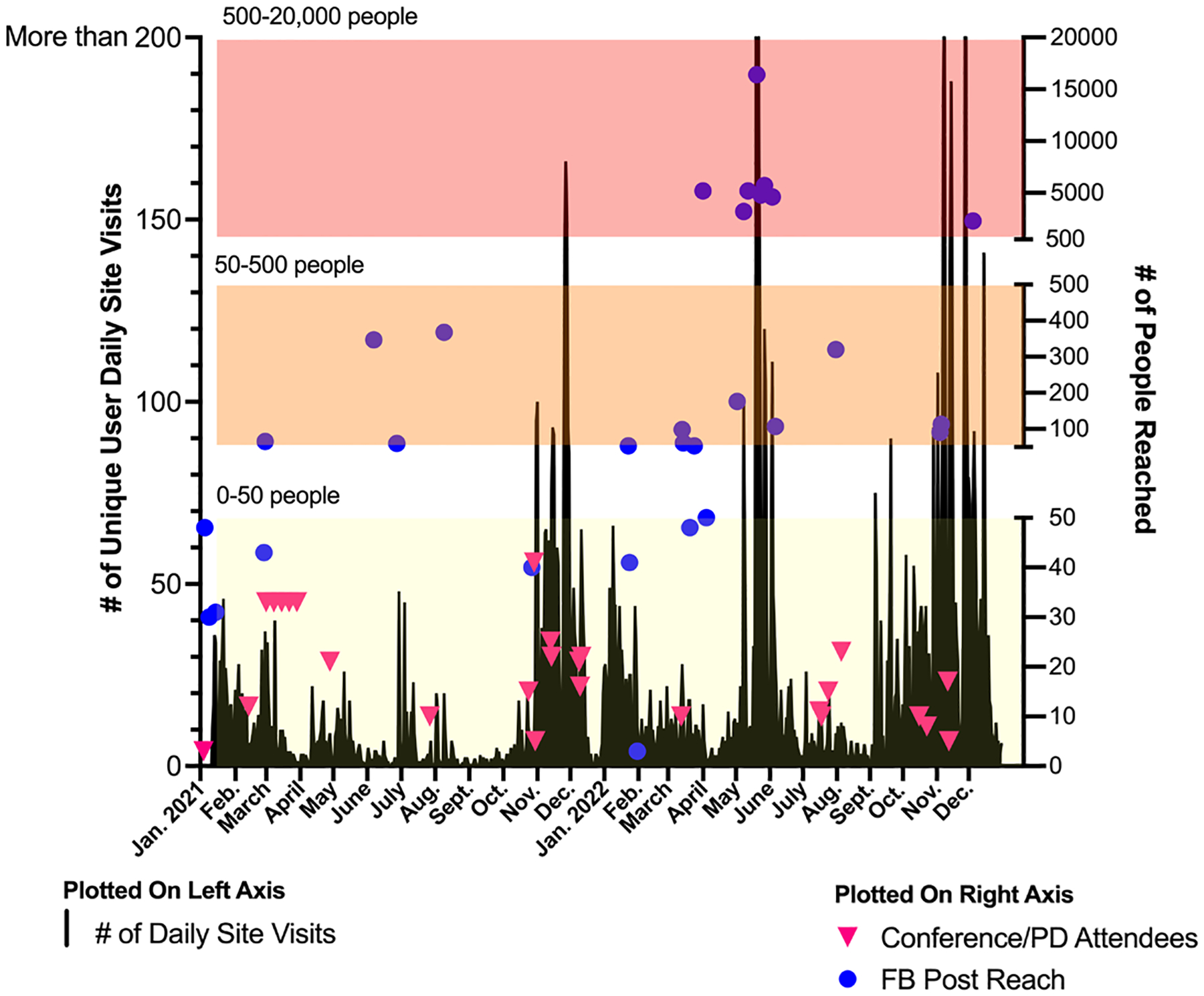
Unique user curriculum site visits with number of people engaged with each event from January 2021-December 2022. Left y-axis is unique daily Curriculum Site visits. Right y-axis plots specific events and the number of people reached with each kind of event.

**Figure 4.**
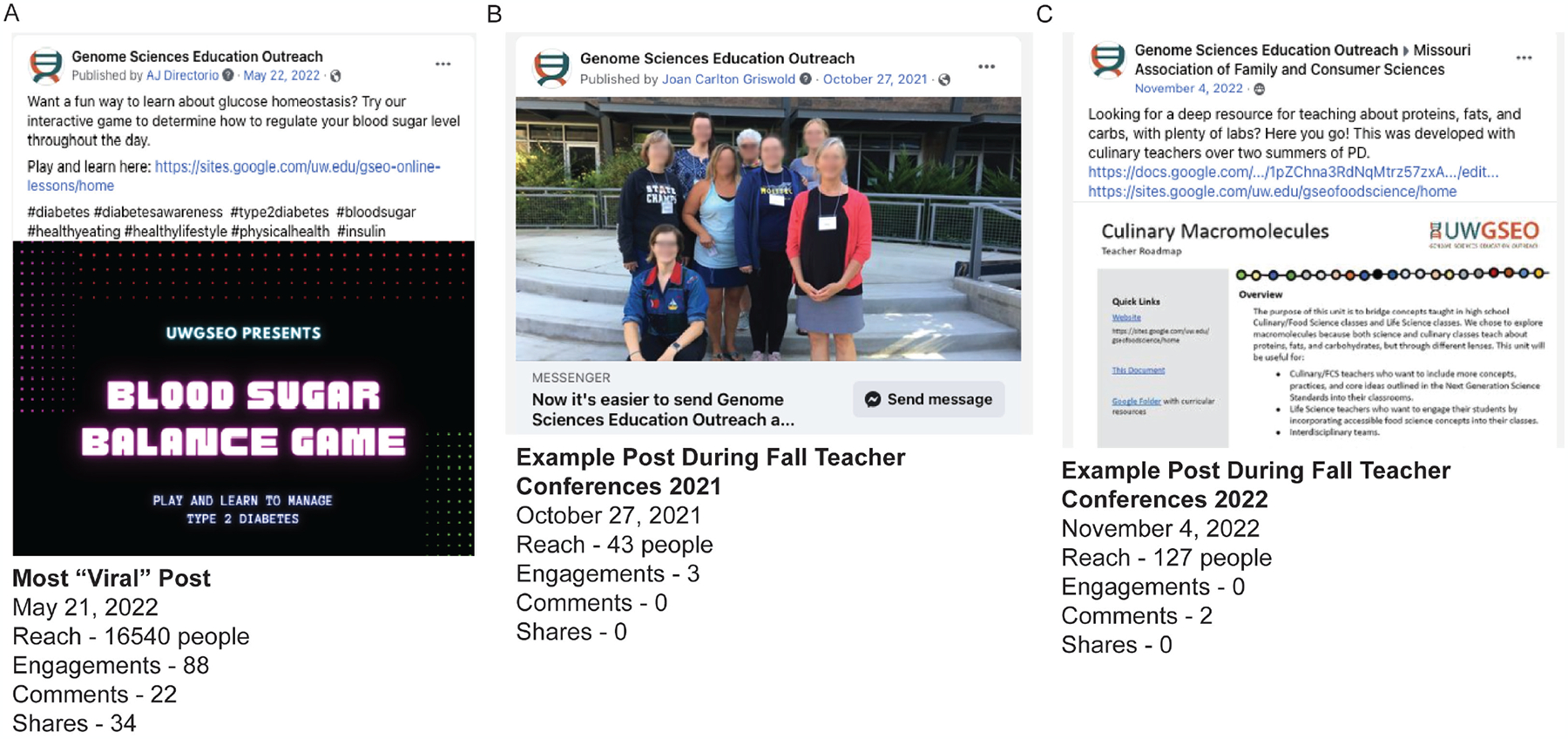
Comparison of social media post performance on Facebook. A) Most viral post promoting the Blood Sugar Balance Diabetes Education Webgame. B) Example post from Oct.-Nov. 2021. C) Example post from Oct.-Nov. 2022.

Overlaying the specific dates of professional development sessions (in-house, online, and at national conferences) and FB posts onto the daily site visitations, demonstrates the specific dates and the number of people reached with each PD sessions or FB posts (Figure 3). Daily site visits were plotted on the left y-axis, with the number of people attending each PD session or reached with each FB post plotted on the right y-axis. PD sessions, whether online or in person, engaged a small number of people with each session (5–35 individuals) and was correlated with bursts of site visits at the time of engagement. Before the launch of the FB campaign in May 2022, the biggest bursts in site visits corresponded with PD sessions in Oct.-Dec 2021 around the time of presenting at teacher conferences like NSTA, NABT, Washington state science teachers association (WSTA), and Washington Family and Consumer Sciences (WA-FACS). FB posts before the start of the campaign reached fewer than 50 people, with the occasional spike in the 100–400 range. During the FB campaign in May 2022, FB posts were reaching upwards of 500–20,000 people and driving a dramatic increase in daily curriculum site visits (100–573) (Figure 3). On FB analytics, the number of “shares” on posts were substantially increased during the campaign, with one post that promoting the educational web-game Blood Sugar Balance going “viral”, driving 573 site visits after reaching 16,400 people on Facebook (Lesiak and Griswold, 2023) (Figure 4A). This dramatic increase was likely due to the increased post engagement, comments, likes, and shares.

Even after the FB campaign and the work with the Communications Coordinator was over in mid-June 2022, FB posts continue to reach more people. Comparatively, a post made in Oct. 27th, 2021 only reached 43 people, while a post made around the same time on Nov. 4th 2022 reached 127 (Figure 4B and 4C). These posts were the only posts made during the annual period of presenting at teacher conferences, and neither were linked towards any of the curricular content being disseminated during those time periods. These FB posts during the Oct.-Nov. teacher conference season were unlikely to drive curriculum site traffic on their own, but were likely the result of teacher use at a conference or driven by teachers actually using the curriculum in their classrooms.

Interestingly, total curriculum site visits went up in Oct.-Dec. 2022 relative to the same period of 2021 (Figure 2 and 3). While not conclusive evidence of the effectiveness of the FB ad campaign, it is unlikely that this increase in curriculum site visits is related exclusively to PD sessions in the Fall of 2022, due to low teacher turnout at each PD session (<20). Instead it is likely a synergistic increase in teacher usage based on the 2021 PD and FB ads the previous year. It is expected that teachers will visit the GEMNet curriculum site more during the following school year in order to prepare to teach the curriculum if they had previously been engaged at conference workshops or learned about the curriculum from a Facebook post.

**Figure 5.**
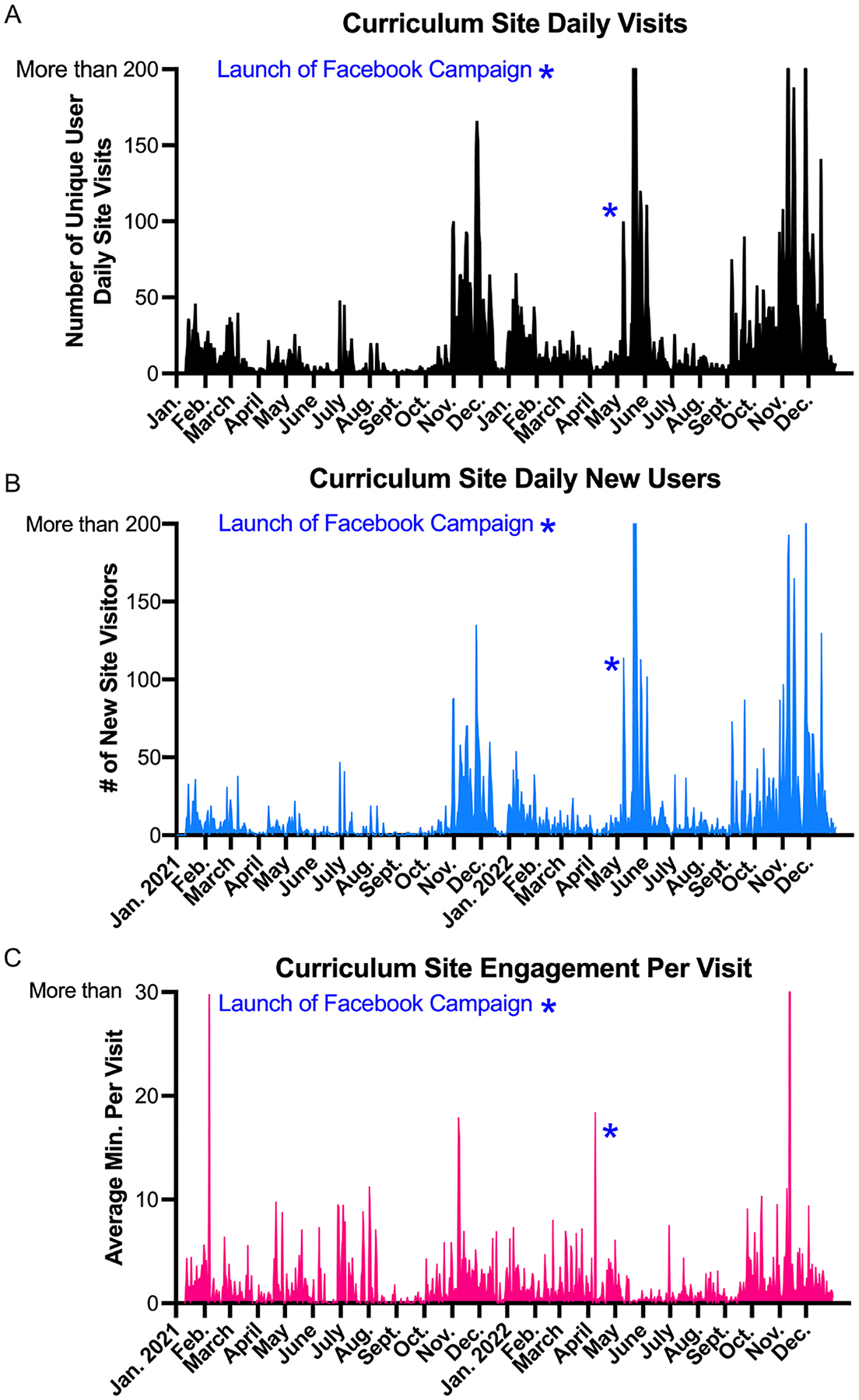
Google Analytics data from Jan. 2021-Dec. 2022 for Curriculum Site. A) Number of unique user visits per day. B) Number of new site visitors per day. C) Average minutes spent on Curriculum Site per visit.

Notably, curriculum site visits are not conclusive evidence that visitors are actually engaging with the site or using any of the lesson resources. Comparing site visits from new users to the average length of engagement per visit, daily site visits and new users track together (Figure 5 A–C). This is likely because the PD and FB posts were routinely reaching potential new users. Unfortunately, Google Analytics was not set-up to identify the factors driving returning user activity, as this would have helped to better understand how teachers were engaging with the curriculum site. New site visitor and return user data can be confounded by the use of Virtual Private Networks (VPNs), making some returning users look like new site visitors every time they visit the site.

Interestingly, during the FB campaign in May 2021, the average length of time each user engaged with the curriculum site was noticeably lower than periods of direct teacher engagement during PD sessions (Figure 4C). Reasons for this drop in time spent on the curriculum site could be: A) Site visitors from FB ads are interested in a quick overview but do not spend time to actually peruse curriculum resources, or B) During PD sessions, teachers use the website to engage with materials online and inherently spend more time on the website, or C) Teachers download materials to engage with offline, or bookmark content to return to at a later time. This difference in engagement time is likely a combination of these factors, but the analytics system was insufficient to identify the true cause.

Regardless of the average time spent engaging with the curriculum during each site visit, the primary goal was to get the curriculum resources in front of as many teachers as possible. Even if teachers don’t spend a lot of time engaging with the site after a FB post, at least they are aware of the materials available, and have the ability to return to the curriculum if and when they want to use it in the future.

## DISCUSSION

### Creating a Science Communication Training Pipeline to Enhance Dissemination.

Often academic training spaces are siloed in a way that limits career development opportunities for trainees. It can be very difficult to establish interdepartmental collaborations when program managers have limited capacity, and trainee scholarship objectives are prioritized over extra-curricular activities. The need for modernized communication strategies in STEM fields is deeply important, evidenced by the spread of misinformation during the COVID-19 pandemic and surrounding a myriad of other public health and environmental crises (Fischhoff, 2013; Matta, 2020). Science communication degrees and certificates are increasingly being offered at universities to meet demand for more specialists in the field. Investing in optimal strategies to connect STEM outreach programs with teachers, and their students, is essential to develop foundational science literacy and competency among the next generation of citizens and future scientists.

STEM outreach and science communication can be enhanced through interdepartmental collaborations to provide interdisciplinary training pipelines during graduate and undergraduate education. Integrating training in communications with STEM Outreach programs highlights a meaningful opportunity for enhancing resource dissemination to teachers. In future grants, GSEO will integrate the CommLead collaboration into all of their proposals to create a pipeline for communications trainees to gain experiences in science communication and enhance the dissemination of GSEO’s educational resources.

### Developing and Measuring Digital Dissemination Strategies for STEM Outreach Programs.

Dissemination is essential for education outreach programs to reach a broader teacher population in an affordable, equitable, and sustainable way. Face-to-face teacher engagement provides a platform for deeper connection around educational resources, and a teacher that adopts resources after an in-person training can have a substantial impact on students for years to come. Unfortunately, only teachers with capacity to spend the time and/or money to travel or attend sessions will have access to such materials made available exclusively through conference presentations and in-person PD sessions. For education outreach programs on a budget and with limited staff capacity, the expenditures to attend and present at conferences is substantial, and teacher attendance can be highly variable and unpredictable. It can be disheartening to invest the time and money to travel to and present at a conference only to have poor attendance at your session. Regardless of its limitations, in-person engagement with teachers through PD trainings will continue to be an excellent approach for resource dissemination; however, there is great potential for a more diversified dissemination approach to connect with a broader and more inclusive population of teachers.

With a relatively small investment in social media and digital outreach, GSEO was able to enhance the reach of their educational resources beyond the reach of their traditional dissemination approach. Investment in staff to generate posts and connect with teacher organizations on social media led to spikes in website traffic and better networking with teacher organizations on a national level. Greater investment in digital marketing and social media could have a considerable impact on the reach of education outreach programs. The current study could provide a framework and rationale for other education outreach programs to similarly invest in digital dissemination through social media campaigns.

Notably, this case study of the ad hoc implementation and evaluation of GSEO’s digital communication and social media strategy has notable limitations. Perhaps the biggest factor to highlight was a lack of sophistication regarding the use of Google Analytics to measure site traffic. The primary goal was to drive more traffic to the GEMNet website with social media posts. The spikes in traffic, although correlated with social media posts, could have been affected by teachers returning to the site after successful engagement during a different professional development training. GSEO was unable to measure the return user rate or curriculum usage data by visitors engaged by social media posts. Therefore, it is not certain that the increased site visitations led to teachers actually using the curriculum. Additionally, it is unclear whether the increase in site users in the Fall of 2022 relative to 2021 was a result of in-person PD workshops, messaging through teacher listservs, or due the social media posts the prior year.

For future research into the effectiveness of digital dissemination, programs would be advised to get better baseline analytics for a more effective analysis of their dissemination campaigns. Prior to the creation of the educational resource user site in early 2020, most of GSEO’s resources were hosted as pdf-links on their outdated website (https://gsoutreach.gs.washington.edu/). Website analytics for the GSEO website were never established prior to the initial curriculum dissemination phase in 2018, and until 2020 most of the educational resources were disseminated in the form of a three-ring binder at in-person PD sessions combined with follow-up surveys, making it difficult to track teacher usage with precision. In addition to tracking visits to the GSEO website as teachers respond to FB posts, future research would include a measure of how often a resource is “shared” by teachers on FB, as this suggests a higher level of perceived quality by teachers (Baas et al., 2022; Clements and Pawlowski, 2012). Ideally, the ability to measure and track teacher’s usage of the educational resources would have been integrated into the dissemination strategy in order to provide better analytics.

## Conclusion.

GSEO’s small group lacked the knowledge and capacity to drive their social media presence alone, and resources for establishing and maintaining their social media presence as part of their dissemination strategy was not included in the initial grant application. Investing in a communications coordinator through the partnership with CommLead allowed for enhanced engagement with teacher organizations, the maintenance of a consistent social media presence online, and furthered the program’s goals of curriculum dissemination. Additionally, it offered a real-world experience in science education to a graduate student, and built a bridge between academic departments. Data from the described collaboration highlights the power that a social media manager can have on the visibility of education outreach programs.

## ABBREVIATIONS

CC: Communications Coordinator
FB: Facebook
GEMNet: Genes, Environment and Me Network
GSEO: Genome Sciences Education Outreach
PD: Professional Development
SEPA: Science Education Partnership Award
UW: University of Washington
VPN: Virtual Private Network
WA-FACS: Washington Family and Consumer Sciences
WSTA: Washington State Science Teachers Association
